# Synergistic effects of *CP-25* and 5-fluorouracil on the hepatocellular carcinoma in *vivo* and in *vitro* through regulating activating mitochondrial pathway

**DOI:** 10.7150/jca.54702

**Published:** 2022-01-04

**Authors:** Wei Chang, Xiao-Pei Jiang, Shuai Jin, Pei-Pei Li, Sha-Sha Song, Ping-Fan Yuan, Wei Wei, Jing-Tao Lu

**Affiliations:** Institute of Clinical Pharmacology, Anhui Medical University, Key Laboratory of Anti-inflammatory and Immune Medicine, Ministry of Education, Hefei 230032, Anhui Province, China

**Keywords:** *CP-25*, Hepatocellular carcinoma, Diethylnitrosamine, Apoptosis, C57BL/6J

## Abstract

Paeoniflorin-6'-O-benzene sulfonate (*CP-25*) has therapeutic potential for the treatment of hepatocellular carcinoma (HCC). 5-Fluorouracil (5-Fu) has been a conventional chemotherapeutic agent for HCC. Unfortunately, the nonspecific cytotoxicity and multidrug resistance caused by long-term use limited the clinical efficacy of 5-Fu. This study was aimed to investigate whether the combination of *CP-25* and 5-Fu could generate synergistic effect in inhibiting HCC. The experiments on the diethylnitrosamine (DEN) -induced mice showed that compared with applying single drugs, the combination of *CP-25* and 5-Fu presented stronger inhibition in tumor nodule and volume. Meanwhile, CP-25 and 5-Fu activated the intrinsic mitochondrial apoptosis pathway induced by P53, inhibited anti-apoptotic B-cell lymphoma (Bcl-2), induced the pro-apoptotic Bcl-2-associated X protein (Bax), Cytochrome-C and caspases. In addition, the synergistic effect was also validated in Bel-7402 and HepG-2 cells in *vitro*. This research not only provides a novel and effective combination strategy for the therapy of HCC but also provides an experimental basis for the development of *CP-25* and 5-Fu compound preparation.

## Introduction

Hepatocellular carcinoma (HCC) is the leading cause of cancer-related death worldwide with very poor prognosis and high mortality 1, 2. In spite of the advances of classical therapies, such as surgery, transplantation, use of radiofrequency and transarterial embolization, the prognosis of this neoplasm has not considerably improved over the past few years 3. Therefore, development of new anticancer drugs and adjuvants, including herbal medicines, is essential for treatment patients with HCC.

5-Fluorouracil (5-Fu) was one of the first-line chemotherapy drugs for the treatment of malignant tumors including liver, breast and other digestive system tumors 4-6. However, the clinical application of 5-Fu was limited due to its inevitable toxicity to normal cells and multidrug resistance caused by long-term use 7, 8. Recent studies have found that 5-Fu combined with natural drugmonomers can reduce the dose of 5-Fu and increase the therapeutic effect, such as curcumin (CU), huaier, forbesione. Thus, there is an urgent need to identify new chemosensitizers to treat patients with HCC.

**Fig 1:**
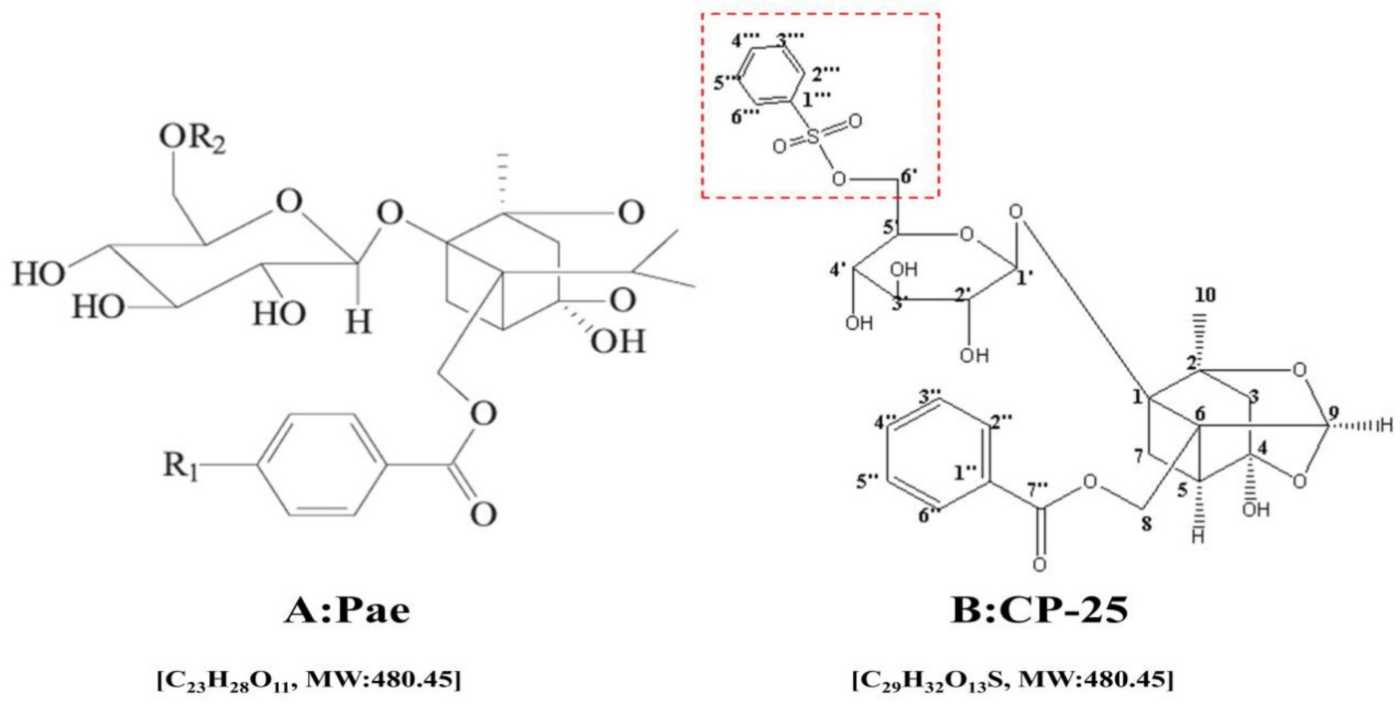
The chemical structure of Pae (A) and *CP-25* (B)

Paeoniflorin-6'-O-benzene sulfonate (*CP-25*, C29H32O13S, MW: 620.62, Fig. 1.) a novel ester derivative of paeoniflorin (pae, C23H28O11, MW: 480.45, Fig. 1.) which has better bioavailability and higher anti-inflammatory immune regulatory effects than pae 9. Pharmacologically, pae has long been used as an herbal medicine to treat arthritis, systemic lupus erythematosus (SLE), fibrosis, hepatic granuloma, diabetic nephropathy (DN) and cancer 10-15. Meanwhile, the anti-inflammatory and immunoregulatory effects of *CP-25* have been verified in many studies 16-18. However, its anti-tumor effect is still unclear. Our previous investigations suggest that *CP-25* inhibited HCC cell proliferation and induced cell apoptosis in a dose- and time-dependent manner. However, there is no any information about the chemosensitization effect of *CP-25* until now.

In the present study, we utilize the DEN-induced HCC mouse model, and human HCC HepG2, Bel-7402 cells to explore the possible chemosensitization effect of *CP-25* to potentiate the antitumor effect of 5-Fu in *vitro* and in *vivo*, and found that these two agents act synergistic anticancer activity. These findings may offer a promising new approach in the effective treatment of human HCC.

Resistance to cell death is a distinctive characteristic of cancer 19. Apoptosis is a strictly controlled mechanism of cell suicide and can be triggered by different pathways that depend on the initial stimulus 20. The extrinsic pathway requires the activation of transmembrane receptors by soluble death ligands, such as Fas ligand (Fas-L), to initiate a cascade of events and finally stimulate caspase 3 pro-death activity 21. The intrinsic pathway involves the mitochondrial release of Cytochrome-C, allowing apoptosome complex formation and consequently procaspases activation. The permeabilization of mitochondrial membrane increases the translocation of Cytochrome-C to cytosol, and this process is regulated by the Bcl-2 family of proteins 22. The cascade of apoptosis signal transduction begins by the action of initiator caspases that are recruited and activated by autocatalytic processing. Among these caspases, Caspase- 8 and 9 are the main initiators of programmed cell death; caspase 8 is stimulated in response to extrinsic death ligands, while caspase 9 is necessary for the activation of executor Caspase-3 23. Targeting both the intrinsic and extrinsic pathways reduces the growth of different tumour types, and a large number of studies demonstrate that different drugs alone or in combination enhance apoptosis in cancerous cells, including HCC 24.

Mouse models of HCC are the most basic condition of HCC experiments. The mouse models of HCC include spontaneous, induced, portability and genetically modified animal models of HCC. Diethylnitrosamine (DEN) is the main chemical agent that has potent hepatocarcinogenic effect that is used to induce HCC in an experimental mice model. Accordingly, we selected this model to investigate the anti-tumor effect of *CP-25* and 5-FU on DEN-induced HCC mice and explore the possible underlying molecular mechanisms.

## Materials and Methods

### Drugs and reagents

*CP-25* was purchased from Institute of Clinical Pharmacology, Anhui Medical University, degree of purity ≥98% (Hefei, P.R. China). 5-Fu was purchased from Shanghai Xudong Haipu Pharmaceutical Co. (Shanghai, P.R. China). Diethylnitrosamine (DEN) was purchased from Sigma degree of purity 98% (St, Louis, MO, USA). P53 and β-actin antibodies were obtained from Santa Cruz Biotechnology (Santa Cruz, CA, USA). Cytochrome C were purchased from Abcam (Cambridge, England, UK). Caspase-9 were purchased from Cell Signaling Technology (Colorado, USA). B-cell lymphoma (Bcl-2) and bcl-2-associated X protein (Bax) were purchased from Zhongshan Goldenbridge Biotechnology (Beijing, P.R. China). MMP-2 and MMP-9 were purchased from Biosynthesis Biotechnology (Beijing, P.R. China). The kits for ALT, AST, ALP and AFP were purchased from Nanjing Jiancheng Bioengineering Institute (Nanjing, P.R. China). All other reagents were purchased in the purest from available.

### Animals and treatment

Bel-7402, HepG-2 cells were obtained from Shanghai cell bank of China, and the cells were cultured in RPMI 1640 medium supplemented with 100 units/mL of penicillin-streptomycin and 10% fetal calf serum at 37℃ in a 5% CO2 incubator (HEPA class100 Thermo company). Medium was replaced 3 times a week. Cells were used in the exponential growth phase for all of the experiments.

C57BL/6J mice, weighing 4±6 g, were obtained from the Laboratory Animal Centre of Anhui Medical University and maintained at an animal facility under pathogen-free conditions. All experiments were approved by Ethics Review Committee for Animal Experimentation of Institute of Clinical Pharmacology, Anhui Medical University. After acclimating for 1 day, mice were randomly divided into five groups, including control group, DEN-treated-, *CP-25* (70 mg/kg)-, 5-Fu (20 mg/kg)-, 20 mg/kg 5-Fu + 70 mg/kg *CP-25* groups. Excepting the control group, mice were intraperitoneally injected with DEN (2 mg/kg) at 14 d persisting 44 weeks. On the 16th week after the intraperitoneally injection with DEN, the administration group was given the corresponding test substance persisting 8 weeks. On the 44th week, the body weight of mice was measured, the blood sample was taken by removalling eyeball, liver specimens were removed and weighed. Macroscopically visible liver tumors and nodules greater than approximately 1 mm in diameter on liver surface were recorded. The right lobe of each liver was fixed in formalin solution for histopathological examination. The remaining liver tissues were stored at -80 °C for biochemical and western blot assays.

### Histopathology Analysis

For histological examination, a specimen of liver was trimmed and fixed in 10% formalin immediately and embedded in paraffin. Serial 3 um sections were stained with hematoxylin and eosin (H&E). The changes of liver pathological and the extent of liver tumors were observed and diagnosed under a microscope (× 200 magnification).

### Measurement of serum AST, ALT, ALP and AFP levels

Serum was separated from the mice blood in a centrifuge under 654 g for 20 min at 4 °C. The serum level of AFP, AST, ALT and ALP was measured using commercially available kits.

### Calculation of liver and spleen index

Liver and spleen was removed and weighed. Liver and spleen index was calculated as liver weight divided by body weight (mg/g).

### Western Blot Analysis

Liver fragments were lysed in RIPA (1% NP-40, 0.5% Na-deoxycholate, 0.1% SDS, 1 mM PMSF and complete protease inhibitor mix in PBS) buffer. The homogenate was incubated on ice for 30 min, and finally, the samples were centrifuged at 14,000 g for 20 min at 4°C. The supernatant fraction was recollected and stored at -80°C in aliquots until use. Protein concentration was measured by the BCA assay. Equal amounts of protein (15-30 μg) were separated by 10-12% sodium dodecyl sulfate (SDS) polyacrylamide gel electrophoresis and then blotted on polyvinylidene fluoride membranes (PVDF membrane, Millipore, USA). The membranes were then blocked with 5% non-fat dry milk in phosphate buffered saline buffer containing 0.05% Tween 20 (TPBS) for 2 h at room temperature and probed overnight at 4°C with primary antibodies of rabbit monoclonal anti-P53, MMP-2, MMP-9, Cytothrome C, cleaved-caspase-9, Bcl-2 and mouse monoclonal anti-Bax, β-actin at 1:200-1:1,000 dilution with WB anti-body diluent. After washing with 0.05% Tween 20-PBS, the membranes were incubated with corresponding secondary antibody (1:70000) at 37 °C for 2 h. The membranes were washed and detected by measuring the chemiluminescence of the blotting agent after exposure of the filters on films. Finally, the densities of the bands were quantified with a computerized densitometer (ImageJ Launcher, Broken Symmetry Software). Equivalent protein loading and transfer efficiency were verified by staining for β-actin.

### Immunohistochemistry

Paraffin-embedded tissue samples were cut into 3 um thin slices and baked in the constant 60 °C temperature box for 2 h. After deparaffinization with Neo-Clear (Merck), endogenous peroxidase was blocked by immersing slides in 2.5% H_2_O_2_ diluted in pure methanol. Unspecific staining was blocked by incubation with goat serum diluted in Tris-buffered saline pH 7.4. Then slices were stained with P53 (1:100), Caspase-9 (1:200), Cytochrome-C antibody (1:250), Bcl-2 (1:100) and Bax (1:100) antibody overnight at 4 °C. After washed the antibody, the slices were incubated with second antibodies at room temperature for 20 min. And then, the slices were stained with DAB and hematoxylin to observe the proteins expression under the microscrope. The IOD and area of cells were measured by IPP 6.0 software, and the fluorescence intensity of proteins was expressed as IOD/area.

### Examination of the effects of combination agents on cells

Before the combination effect was tested, the IC50 (the half maximal inhibitory concentration) was determined from the exposure of the drugs including single agent and the combination agents to the HCC cells by CCK-8 assay 25. And then the test was divided into four groups including: control, *CP-25* alone (10^-5^ mol/L), 5-Fu alone (25 μg/ml), 25 μg/ml 5-Fu +10^-5^ mol/L *CP-25.* The apoptosis rate was detected by flow cytometry and the invasion and metastasis ability of HepG2 and Bel-7402 were observed by scratch test and transwell 26. The expression of P53, Caspase-9, Cytochrome-C, Bax, Bcl-2 in HepG2 and Bel-7402, and MMP-2 and MMP-9 in Bel-7402 were assayed by western blot (reference 2.6).

### Statistical analysis

Data are expressed as mean ± standard deviation (S.D.). One-way analysis of variance (ANOVA) was used to determine significant differences among the groups and the student's *t* test was used to analyze comparison between two groups. A value of *P*<0.05 was considered statistically significant. These analyses were performed using SPSS 21.0 software.

## Results

### *CP-25* treatment improved the antitumor effect of 5-Fu in *vivo*

**Fig 2:**
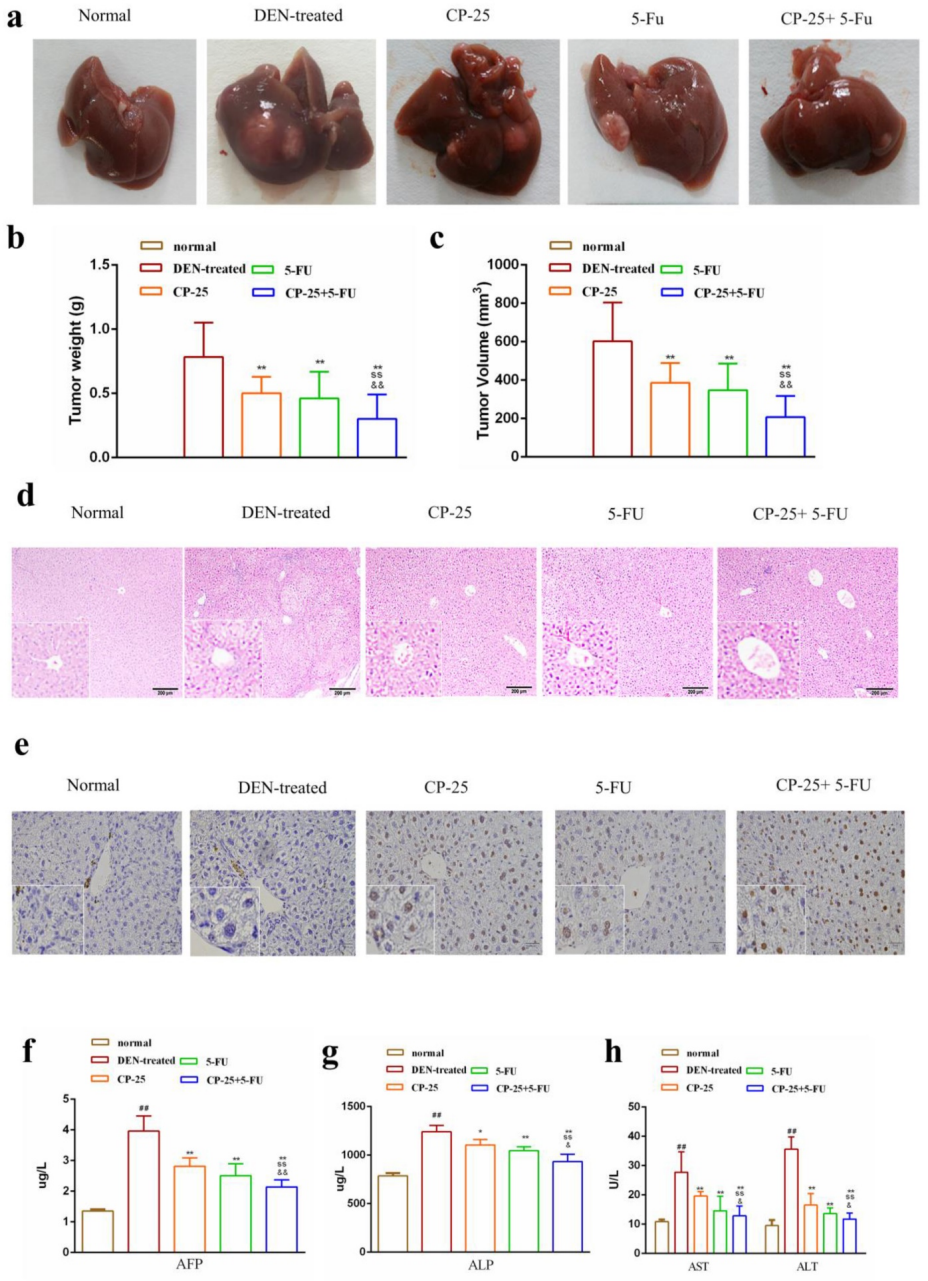
Combination treatment with 5-Fu and *CP-25* inhibited tumorigenicity in *vivo*. a: Representative images of the liver tumors in the different groups. b, c: The final tumor weight and volume in each group after treatment. d, e: Representative images of hematoxylin and eosin (H&E) and tunel staining in each group (original magnification×200). f-h: The levels of ALT, AST, ALP, AFP in each group. All data are expressed as means ± standard deviation. ^#^*P*< 0.05,^##^*P*< 0.01, versus normal group; ^*^P< 0.05, ^**^P< 0.01, versus DEN-treated group, ^s^*P*< 0.05, ^ss^*P*< 0.01, versus CP-25 alone group; ^&^*P*< 0.05, ^&&^*P*< 0.01, versus normal group versus 5-Fu alone group.

We investigated the effect of *CP-25* and 5-Fu on HCC in *vivo*, the HCC model was established in C57BL/6J mice. As Fig. 2a-c illustrated, treatment with 5-Fu (20 mg/kg) or *CP-25* (70 mg/kg) alone had little effect on the growth of HCC tumors, whereas the combination of both agents resulted in a significant reduction in HCC tumor weight and volume. The mean tumor weights in different groups were also calculated, and results showed that the tumor weight inhibition rates were 45.89%, 54.21%, and 73.68 % for 5-Fu, *CP-25*, and 5-Fu + *CP-25* treatment groups, respectively (Fig. 2b). The trend of tumor volume inhibition was consistent with tumor weight inhibition (Fig. 2c).

Further, the liver tissues were examined by hematoxylin and eosin (H&E) and tunel staining. The tumor tissue from DEN-treated mice showed compact tumor cells with blue-purple nuclei and pink cytoplasm and less apoptotic cells. In the 5-Fu or *CP-25* treatment groups, the tumor cells were sparse and separated from each other and present numerous apoptotic cells. In 5-Fu + *CP-25* treatment groups, the structure of the tumor tissue was more seriously damaged than that in 5-Fu or *CP-25* alone group, and the nuclei were polygonal and lightly stained (Fig. 2d, 2e). ELISA assay were performed to detect serum AST, ALT, ALP, AFP levels in *vivo*. Co-treatment with *CP-25* and 5-Fu significantly decreased the AST, ALT, ALP, AFP levels (Fig. 2f-h, *P*< 0.05 *vs* 5-Fu or *CP-25* alone).

### The mechanism of synergistic effect of joint group in *vivo*

**Fig 3:**
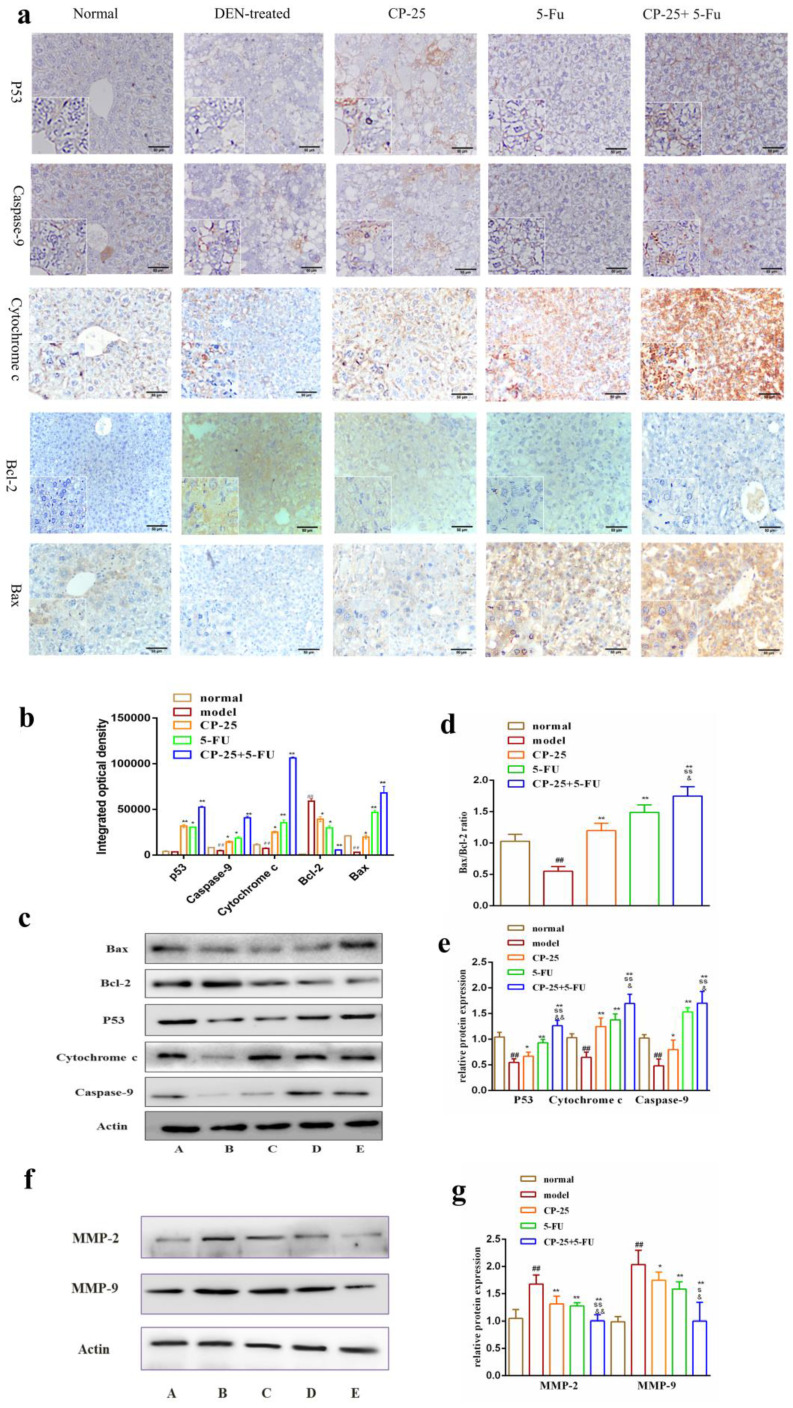
The protein expressions of P53, Caspase-9, Cytochrome-C, Bcl-2, Bax, MMP-2 and MMP-9 in liver issue by Western blotting analysis or immunohistochemistry. a: Representative images of the liver immunohistochemistry in the different groups (original magnification×200). b-d: The expression level of P53, Caspase-9, Cytochrome-C, Bcl-2, Bax in liver issues were detected by western blotting analysis. e-f: The expression level of MMP-2 and MMP-9 in liver issues were detected by western blotting analysis. All data are expressed as means ± standard deviation. A: normal group; B: DEN-treated group; C: *CP-25* group; D: 5-Fu group; E: *CP-25 +* 5-Fu group. ^#^*P*< 0.05,^##^*P*< 0.01, versus normal group; ^*^P< 0.05, ^**^P< 0.01, versus DEN-treated group, ^s^*P*< 0.05, ^ss^*P*< 0.01, versus *CP-25* alone group; ^&^*P*< 0.05, ^&&^*P*< 0.01, versus normal group versus 5-Fu alone group.

After treated with DEN, we observed that anti-apoptotic proteins Bcl-2 and Bcl-xl were significantly decreased and the pro-apoptotic protein Bax was significantly increased in all drug groups compared with DEN-treated group (Fig. 5). In addition,* CP-25* and/or 5-Fu induced the cytochrome C release, resulting in the increase of cytosolic cytochrome C abundance (Fig. 3a-e). Moreover, the cytosolic cytochrome C induced the increasing of caspase-9, which were the executor of apoptosis (Fig. 3a-e). Furthermore, the critical active regulator of p53, was decreased in DEN-treated group, but it was significantly increased in drug groups (Fig. 3a-e). Simultaneously, the effect of regulating proteins in all combined groups was better than that in single groups. The results of immunohistochemistry are in accord with Western Blot (Fig. 3a-e). Furthermore, MMP-2 and MMP-9 were examined in liver after DEN administration and *CP-25* and/or 5-Fu treatment. The expression of MMP-2 and MMP-9 were increased as a result of DEN treatment when compared to normal group. However, *CP-25* and/or 5-Fu reduced the expression level of MMP-9 and MMP-2, especially in combined group (Fig. 3f-g).

### *CP-25* synergistically enhances the antiproliferative effect of 5-Fu in HCC cells

**Fig 4:**
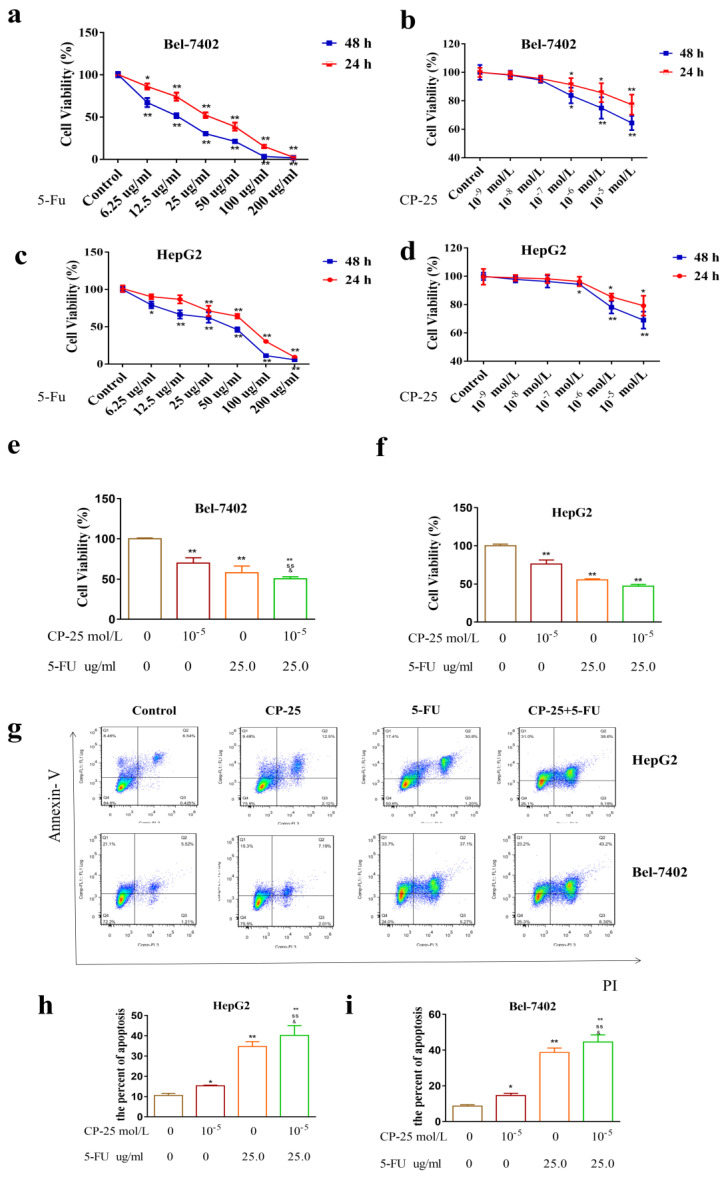
*CP-25* synergistically enhances the antiproliferative effect of 5-Fu in HCC cells. a-d: Cell viability was measured by CCK-8 assay at 24h and 48 h after 5-Fu treatment in Hep-G2 and Bel-7402 cells. e, f: Cell viability was measured by CCK-8 assay at 48 h after 5-Fu and/or *CP-25* treatment in HepG2 and Bel-7402 cells. g-i: The percentage of apoptosis of Hep-G2 and Bel-7402 cells after 5-Fu and/or OMT treatment tested by FLS. All data are expressed as means ± standard deviation. ^#^*P*< 0.05,^##^*P*< 0.01, versus normal group; ^*^P< 0.05, ^**^P< 0.01, versus DEN-treated group, ^s^*P*< 0.05, ^ss^*P*< 0.01, versus *CP-25* alone group; ^&^*P*< 0.05, ^&&^*P*< 0.01, versus normal group versus 5-Fu alone group.

To examine the effect of 5-Fu on cell proliferation and survival, HepG2 and Bel-7402 cells were incubated with increasing concentrations of 5-Fu and cell numbers were counted after 24h, 48 h. Bel-7402 cells performed more sensitive than Hep-G2 cells to 5-Fu treatment (the IC50 value was calculated as 19.03 μg/mL for Bel-7402 and 29.75 μg/mL for Hep-G2 cells, 48 h; Fig. 4a-d).

**Fig 5:**
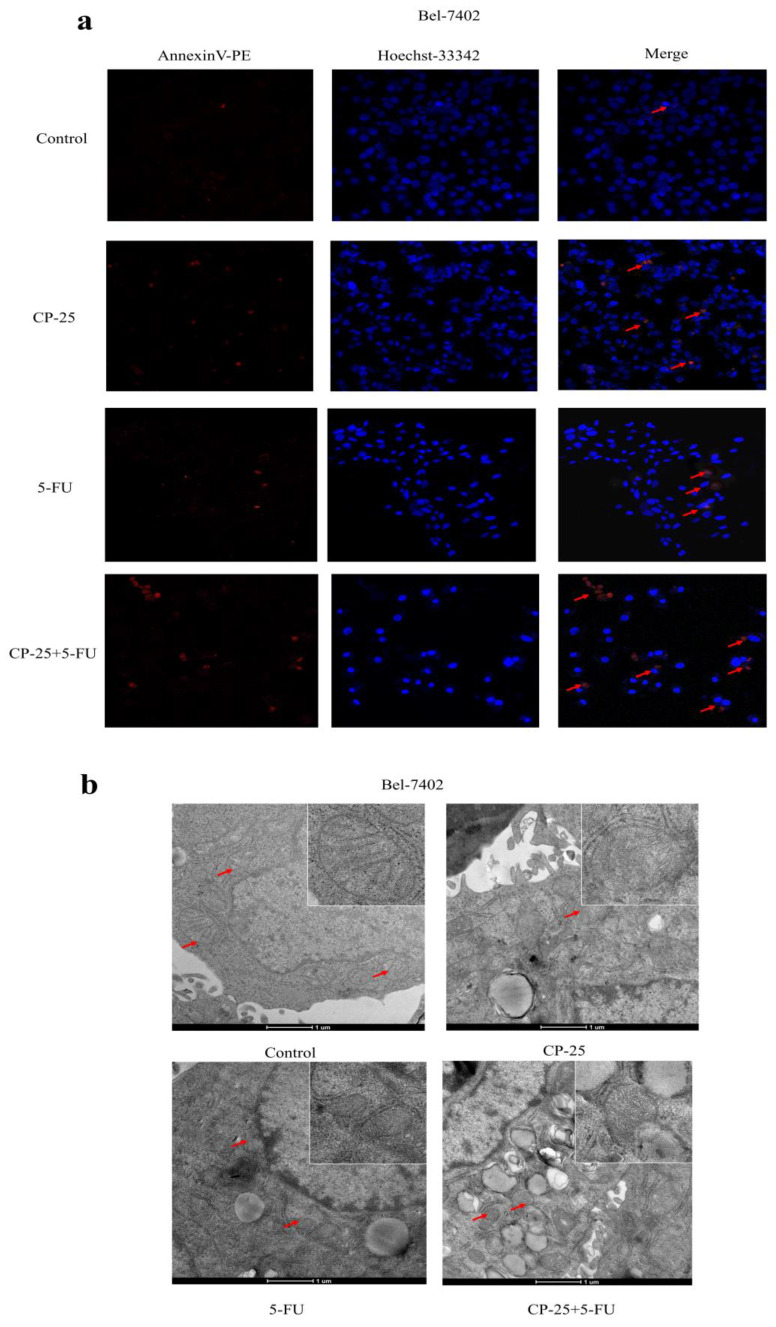
*CP-25* synergistically enhances the pro-apoptosis effect of 5-Fu in Bel-7402. a: Cell apoptosis was measured by Hoechst33342-PE assay at 48 h after 5-Fu and/or *CP-25* treatment in Bel-7402. b: Cell viability was measured by transmission electron microscope (TEM) at 48 h after 5-Fu and/or *CP-25* treatment in Bel-7402.

**Fig 6:**
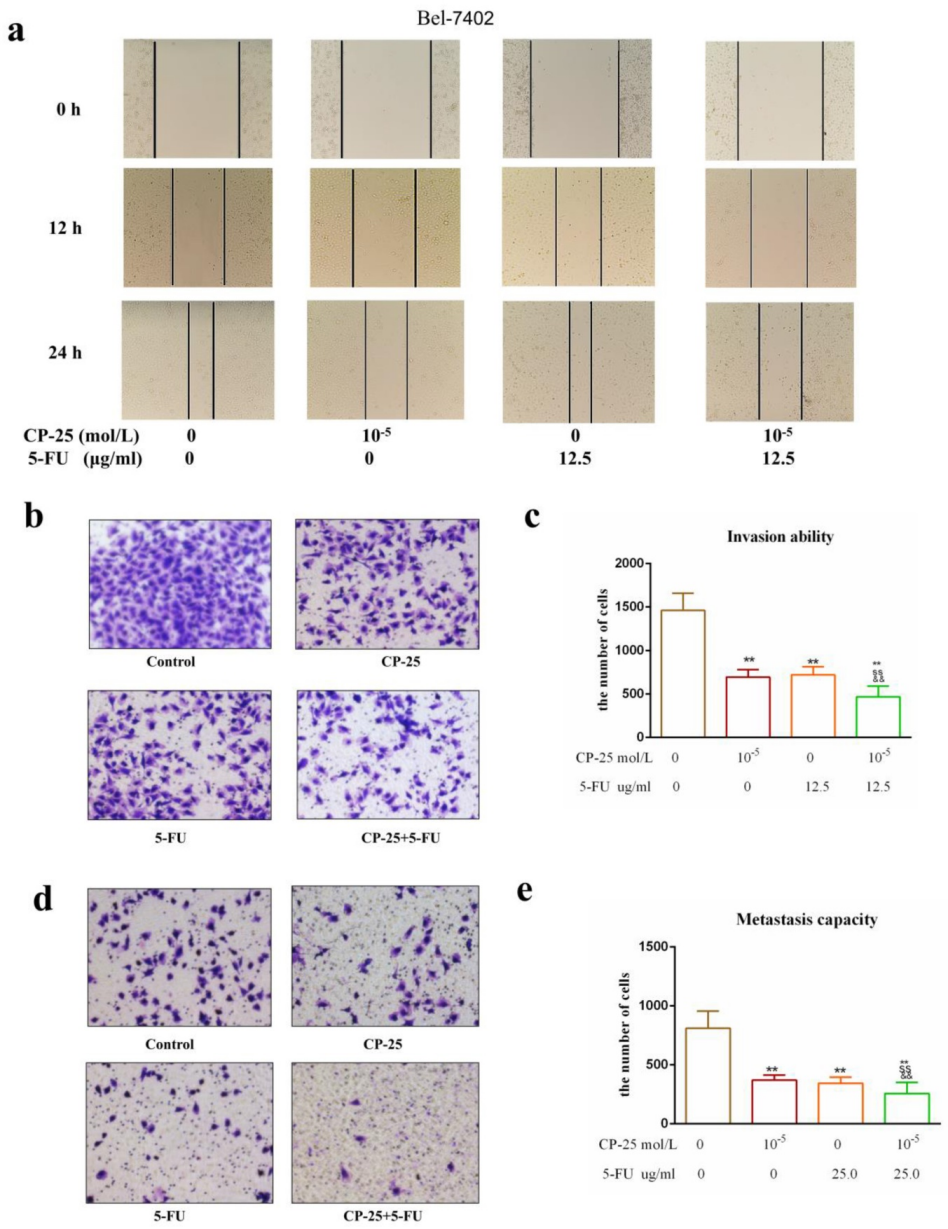
*CP-25* synergistically enhances the invasion and metastasis abilities of 5-Fu in HCC cells. a: Cell invasion ability was measured by scratch assay at 0-24 h after *CP-25* and 5-FU alone or in combination treatment in Bel-7402 cells. b-e: Cell invasion and metastasis abilities were measured by transwell assay at 24 h after *CP-25* and 5-FU alone or in combination treatment in Bel-7402 cells. All data are expressed as means ± standard deviation. ^#^*P*< 0.05,^##^*P*< 0.01, versus normal group; ^*^P< 0.05, ^**^P< 0.01, versus DEN-treated group, ^s^*P*< 0.05, ^ss^*P*< 0.01, versus *CP-25* alone group; ^&^*P*< 0.05, ^&&^*P*< 0.01, versus normal group versus 5-Fu alone group.

However, *CP-25* inhibited Hep-G2 and Bel-7402 cell proliferation in a dose-dependent manner (the highest concentration of *CP-25* currently used in the laboratory is 10^-5^ mol/L, the inhibition rates of HepG2 and Bel-7402 cells after treated *CP-25* (10^-5^ mol/L) 48h are 69.04% and 64.42% respectively; Fig. 4a-d). When co-treated cells with 5-Fu (12.5 μg/mL) and *CP-25* (10^-5^ mol/L) simultaneously, the combined treatment had a stronger inhibitory effect on cell proliferation than either drug alone (Fig. 4e-f). To further validate the synergistic effect of *CP-25* and 5-Fu, the FLS was used to analyze the cell apoptosis of the single and combined treatment. The percentage of apoptotic for 5-Fu + *CP-25* treatment Hep-G2 cells was 35.78 ± 6.03% and Bel-7402 cells was 43.06 ± 5.72% under the applied dosages (Fig. 4g-I, 5a). For Bel-7402 cells, the combination of 5-Fu (12.5 μg/mL) and *CP-25* (10^-5^ mol/L) displayed the best synergistic inhibition capacity, which was selected for further studies. To further explore the mechanism of *CP-25* and 5-Fu on HCC cell, transmission electron microscope (TEM) was performed. As show in Fig. 5b, 5-Fu, *CP-25*, and *CP-25* + 5-Fu group all appeared mitochondrial shrinkage and varying degrees of crest damage in comparison to untreated group. Furthermore, as scratch test and transwell test shown, 5-Fu, *CP-25*, and *CP-25* + 5-Fu group all decreased both the invasion and metastasis ability in comparison to untreated group (*P*< 0.05 *vs* control group), and the strongest one was observed in *CP-25* + 5-Fu group (*P*< 0.01 *vs* 5-Fu or *CP-25* alone) (Fig. 6a-e).

### The mechanism of synergistic effect of joint group in *vitro*

**Fig 7:**
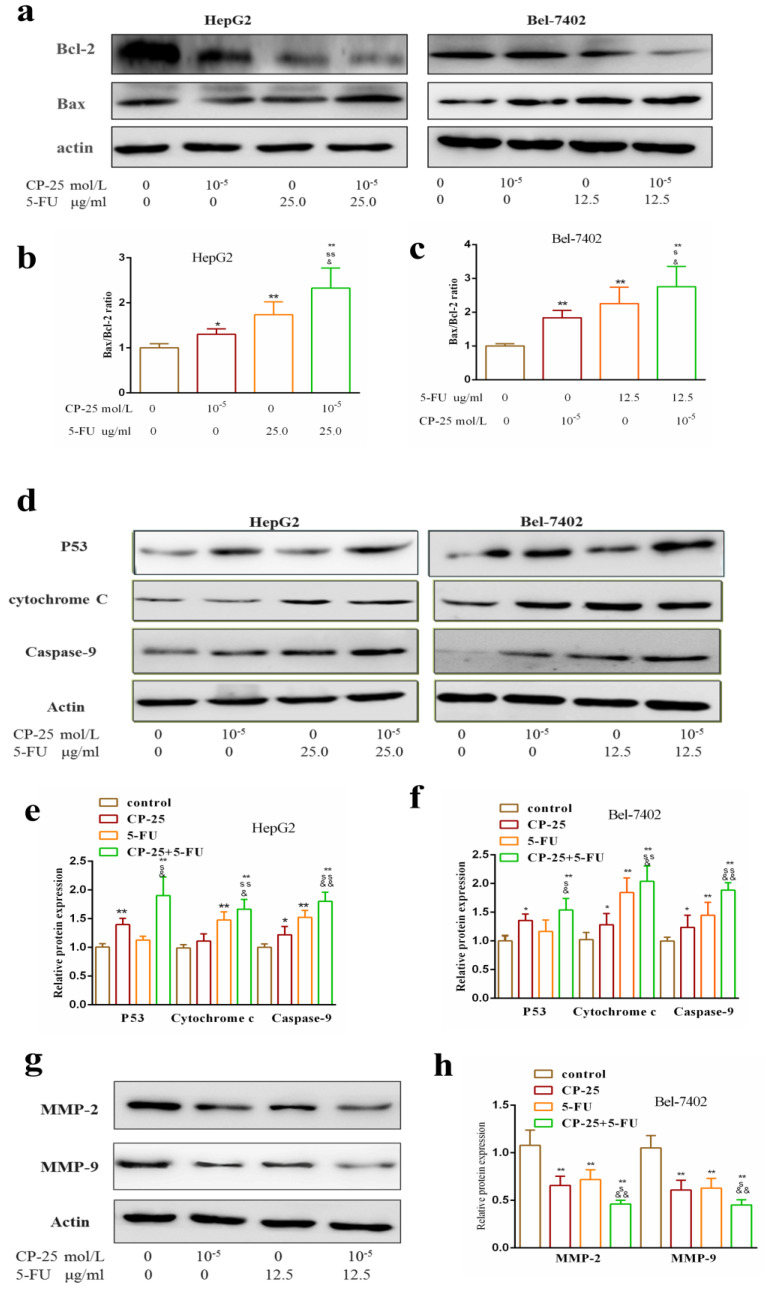
Effects of *CP-25* and 5-FU alone or in combination on the protein expressions of P53, Caspase-9, Cytochrome-C, Bcl-2, Bax, MMP-2 and MMP-9 HCC cells by Western blotting analysis. a-f: The expression level of P53, Caspase-9, Cytochrome-C, Bcl-2, Bax in HepG2 and Bel-7402 at 48 h after *CP-25* and 5-FU alone or in combination treatment. g, h: The expression level of MMP-2 and MMP-9 in Bel-7402 at 24 h after *CP-25* and 5-FU alone or in combination treatment.All data are expressed as means ± standard deviation. ^#^*P*< 0.05,^##^*P*< 0.01, versus normal group; ^*^P< 0.05, ^**^P< 0.01, versus DEN-treated group, ^s^*P*< 0.05, ^ss^*P*< 0.01, versus *CP-25* alone group; ^&^*P*< 0.05, ^&&^*P*< 0.01, versus normal group versus 5-Fu alone group.

Western blotting analysis was performed to analyze the potential mechanism worked in *CP-25* and 5-Fu-triggered apoptosis. The result showed that the combined treatment of both *CP-25* and 5-Fu markedly increased the expression of P53, Cytochrome c, Caspase-9, Bax and decreased the expression of Bcl-2 in HepG2 and Bel-7402 cells (Fig.7a-f, *P*< 0.01 *vs* 5-Fu or *CP-25* alone). Meantime, *CP-25* combined with 5-Fu resulted in a significant decrease of MMP-2 and MMP-9 levels in Bel-7402 cell (Fig.6g-h, *P*< 0.01 *vs* 5-Fu or *CP-25* alone). The results of the study in *vitro* indicated that the combination of *CP-25* and 5-Fu had synergistic effect on inhibiting the HCC cells proliferation, invasion and metastasis, which verified the conclusion of in* vivo* study.

## Discussion

HCC models are essential tools for investigating pathogenesis of HCC. Since the first time building the HCC model, many* in vivo* models have been developed to induce HCC, which included spontaneous, induced, portability and transgenic animal models of HCC 27. As previous remembered, DEN-induced HCC model, similar to human HCC, is the most accepted and widely used experimental models to study hepatocarcinogenesis and screen potential anti-HCC agents 28,29. Here, we used a single injection of nitrosodiethylamine (DEN) to act on C57BL6/J mice, in the hope of successfully establishing the HCC mice model. Our results showed that the levels of serum AFP, ALT, AST and ALP were significantly enhanced in DEN-induced HCC model than in normal group. Meantime, tumor nodules were very obvious in DEN-treated group by visual inspection, which are believed to be very important prerequisites for HCC. Many pathological abnormalities were observed in liver tissues of DEN-induced HCC model group by histological analysis including disordered architecture with a large number of pseudolobules, infiltration of inflammatory cells and abnormal cells with irregular-shaped cytoplasm and enlarged hyperchromatic nuclei. All of these significantly indicated we successfully established the DEN-induced HCC mice model in 44 weeks.

Numerous studies have reported that the extracts of Chinese traditional medicines, such as curcumin, gypenosides, quercetin and oxymatrine could act as chemosensitizer to enhance the efficacy of 5-Fu in HCC cells 26, 30-32. However, whether *CP-25* can become a good chemosensitizer to amplify the effectiveness of chemotherapy in clinic is not clear. Therefore, we evaluated whether *CP-25* could inhibit tumor growth and increase the anticancer effects of 5-Fu in HCC.

In consistent with the previous report 14, 15, *CP-25* significantly suppressed cell proliferation, induces cell apoptosis, and enhances the efficacy of 5-Fu in HCC in *vivo* and in *vitro*. These results indicated that combination administration of *CP-25* and 5-Fu in certain degree may be a potent therapeutic regimen to treat HCC. But its specific mechanism is unclear.

Apoptosis is a process of programmed cell death, which is triggered by changes in the internal and external environment of the cell or death signals. The internal mitochondrial pathway is one of the most important apoptotic pathways 33. The tumor suppressor P53 is an important growth inhibitory and pro-apoptotic protein. Activated P53 can induce the increase of mitochondrial outer membrane permeability by interacting with the Bcl-2 family protein Bax, promote the release of Cytochrome-C, activate caspases and cause cell apoptosis 34. In this study, we found that the P53 level was significantly increased after 5-Fu + *CP-25* treatment in HCC cells and tumor tissue. Caspases as groups of aspartate-specific cysteine proteases regulate the apoptosis induced by different kinds of stimuli 35. Caspase-9 acts as an initiator of Caspase-3 in the mitochondria-dependent pathway. Generally, the caspases regulate apoptosis through DNA fragmentation, chromatin condensation and nuclear fragmentation 36. In the present study, cancer-bearing mice showed reduced expression of caspase-9 as compared to normal control mice. This indicates that DEN injection blocks the apoptosis program through downregulation of caspase-9, but cancer-bearing mice administered with *CP-25* and 5-Fu show significantly increased caspase-9 expression. This upregulation of these genes enhances the apoptotic process. Bcl-2 genes are important regulators for Cytochrome C release from mitochondria and caspases activation. These groups contain both apoptotic and antiapoptotic genes. In our study, DEN treatments upregulated the Bcl-2 proteins in liver of mice as compared to normal mice. Results confirmed that DEN treatment induced cell proliferation and inhibits cell apoptosis in mice. Increased cell proliferation and inhibition of cell apoptosis cause tumor and cancer, whereas in cancer-bearing mice treated with *CP-25* and 5-Fu, Bcl-2 were significantly downregulated as compared to cancer-bearing mice. From these results, it is concluded that *CP-25* and 5-Fu significantly activates the apoptotic process through upregulation of apoptotic genes and downregulation of anti-apoptotic genes. In addition, in *vitro* study, we found that both *CP-25* and 5-Fu could increase obvious P53, Caspase-9, Cytochrome C, BAX, and increase Bcl-2, especially when cells treated with the combination of *CP-25* and 5-Fu, which was consistent with our findings in *vivo*.

The metastasis of cancer cells is a complicated process, including the escape from primary site, degration of ECM (extracellular matrix), invasion into blood and lymph vessel, and reaching new place at last 37. During the process, MMPs play a key role 38. MMP-2 and MMP-9, as the major MMPs, pertain to a zinc-dependent family of endopeptidases involved in a variety of physiological processes 39. In our experiments, the results show that the levels of MMP-2 and MMP-9 in HCC were immensely increased. On the contrary, *CP-25* and 5-Fu can significantly inhibit the expression and activity of MMP-2/-9, which implies that *CP-25* and 5-Fu may through regulating MMP-2/-9 to against the metastatic effect of cancer cells.

## Conclusion

**Fig 8:**
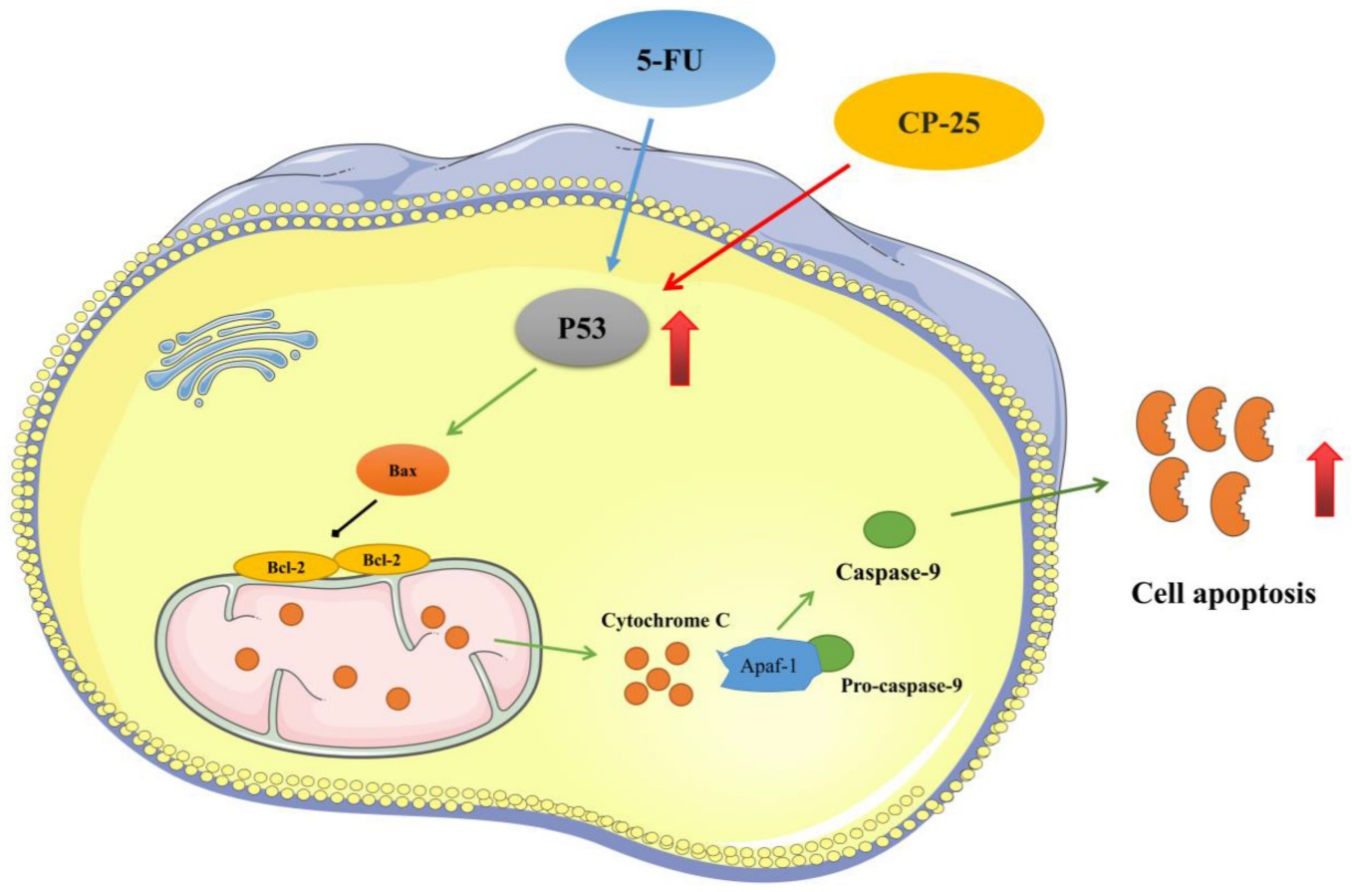
The synergistic effect mechanism of *CP-25* and 5-Fu on HCC

In conclusion, our present investigation confirms that cotreatment of *CP-25* and 5-Fu synergistically inhibited HCC cell proliferation, induced cell apoptosis, and reduced invasion and metastasis in *vitro* and in *vivo*. The synergistic effect of *CP-25* and 5-Fu may be the accumulation of p53 and activation of p53-induced mitochondria apoptosis pathway (Fig.8). Thus, we propose that using *CP-25* and 5-Fu in combination may be as a highly efficient way to achieve antitumor synergism in the clinical treatment of HCC.
